# Identifying resurrection genes through the differentially expressed genes between *Selaginella tamariscina* (Beauv.) spring and *Selaginella moellendorffii* Hieron under drought stress

**DOI:** 10.1371/journal.pone.0224765

**Published:** 2019-11-13

**Authors:** Wei Gu, Aqin Zhang, Hongmei Sun, Yuchen Gu, Jianguo Chao, Rong Tian, Jin-Ao Duan

**Affiliations:** 1 School of Pharmacy, Nanjing University of Chinese Medicine, Nanjing, China; 2 Jiangsu Collaborative Innovation Center of Chinese Medicinal Resources Industrialization, Nanjing University of Chinese Medicine, Nanjing, China; Chinese Academy of Medical Sciences and Peking Union Medical College, CHINA

## Abstract

*Selaginella tamariscina* (Beauv.) spring, a primitive vascular resurrection plant, can survive extreme drought and recover when water becomes available. To identify drought-inducible genes and to clarify the molecular mechanism of drought tolerance, a comparative transcriptional pattern analysis was conducted between *S*. *tamariscina* and *Selaginella moellendorffii* Hieron (drought sensitive). 133 drought related genes were identified, including 72 functional genes and 61 regulatory genes. And several drought responsive reactions, such as antioxidant activity, osmotic balance, cuticle defense and signal transduction were highlighted in *S*. *tamariscina* under drought. Notably, besides peroxidase, catalase and L-ascorbate oxidase genes, DEGs associated with phenylalanine metabolism and polyamine catabolism could be alternative ways to enhance antioxidant ability in *S*. *tamariscina*. DEGs related to soluble carbohydrate metabolism, late embryogenesis abundant protein (LEA) and aquaporin protein (AQP) confirmed that osmotic adjustment could resist drought during desiccation. DEGs involved in xyloglucan metabolic process, pectin metabolic process and cutin biosynthesis may also contribute to drought tolerance of *S*. *tamariscina* by cuticle defense. Drought-responsive genes encoding protein kinases, calcium sensors, transcription factors (TFs) and plant hormones also help to drought resistance of *S*. *tamariscina*. The preliminary validation experiments were performed and the results were consistent with our hypothetical integrated regulatory network. The results of this study provide candidate resurrection genes and an integrated regulatory network for further studies on the molecular mechanisms of stress tolerance in *S*. *tamariscina*.

## Introduction

Plants of the *Selaginella* genus occupy a key phylogenetic position in the evolution of vascular plants [1,2]. Like all non-seed plants, their gametophyte generation is independent, but is dependent on water [3,4]. As one member of the *Selaginella* genus, *Selaginella tamariscina* (Beauv.) spring is a herbal medicine that is commonly used to treat cancer, hepatitis, and diabetes, and is widely used in clinical practice [5–8]. In particular, *S*. *tamariscina* has evolved desiccation tolerance to survive an extreme drought state and is restored as soon as water becomes available[9]. Currently, with the spread of arid and semi-arid areas, screening drought-resistant varieties, revealing their drought resistance mechanism, and developing their potential application value has become a popular research topic. *S*. *tamariscina* is of great theoretical and practical significance in research on drought resistance mechanisms because of its unique resuscitation habits.

Previous studies showed that *S*. *tamariscina* could ameliorate the harm caused by drought via diverse physiological and biochemical reactions. The levels of phytohormones were altered under water stress, for example, the content of abscisic acid (ABA) was markedly increased to regulate several drought-responsive transcription factors (TFs) and the activities of four antioxidant enzymes, including superoxide dismutase (SOD EC:1.15.1.1), peroxidase (POD, EC:1.11.1.7), catalase (CAT, EC:1.11.1.6), and glutathione reductase (GR, EC:1.8.1.7), resulting in reduced oxidant damage induced by reactive oxygen species (ROS)[10]. In addition, increased amounts of soluble sugars were produced to maintain the osmotic balance and cell structure integrity[11]. The proteome analysis of *S*. *tamariscina* revealed that most proteins were downregulated upon dehydration [12]. However, the genetic basis of drought tolerance and the drought-resistant transcriptome of this species remain unclear.

*Selaginella moellendorffii* Hieron belongs to the same genus as *S*. *tamariscina* does and has a very close relationship with *S*. *tamariscina*. in evolution. But the two closely related species of Selaginella differ in their sensitivity to desiccation. *S*. *tamariscina* has evolved desiccation tolerance (DT), whereas *S*. *moellendorffii* is a desiccation-sensitive species that lacks the resurrection trait [13]. Under the same conditions, the water loss rate of *S*. *moellendorffii* was significantly higher than that of *S*. *tamariscina* during dehydration. *S*. *tamariscina* can survive in a dry state, whereas *S*. *moellendorffii* will die when the relative water content (RWC) is less than 40%[13].

The aim of the present study was to conduct a comparative transcriptional pattern analysis on *S*. *tamariscina* and *S*. *moellendorffii*. The gene expression profiles of both species were then analyzed to systematically investigate the classification of differentially expressed genes (DEGs), relative gene expression levels, and drought-inducible genes under water deficit conditions.

To the best of our knowledge, this is the first report of a comparative transcriptional pattern analysis between *S*. *tamariscina* and *S*. *moellendorffii*. Our results will deepen our understanding of the molecular mechanisms of drought tolerance in *S*. *tamariscina*. This study also identified some attractive candidate genes and provided valuable information to improve the drought stress tolerance of other species via genetic engineering.

## Materials and methods

### 2.1 Plant materials and water deficit stress treatments

*Selaginella tamariscina* (Beauv.) spring and *Selaginella moellendorfii* Hieron are common plants which do not require any permission for their manipulation and handling. The study was approved by the College of Pharmacy, Nanjing University of Chinese Medicine.

*Selaginella tamariscina* (Beauv.) spring was collected from Lianyungang, Jiangsu Province, China and *Selaginella moellendorfii* Hieron was collected from Yixing, Jiangsu Province, China. Both plants were identified by Professor Gu Wei (School of Pharmacy, Nanjing University of Chinese Medicine, China) and then cultured in the medicinal botanical garden of Nanjing University of Chinese Medicine under the natural environment.

**Fig 1:**
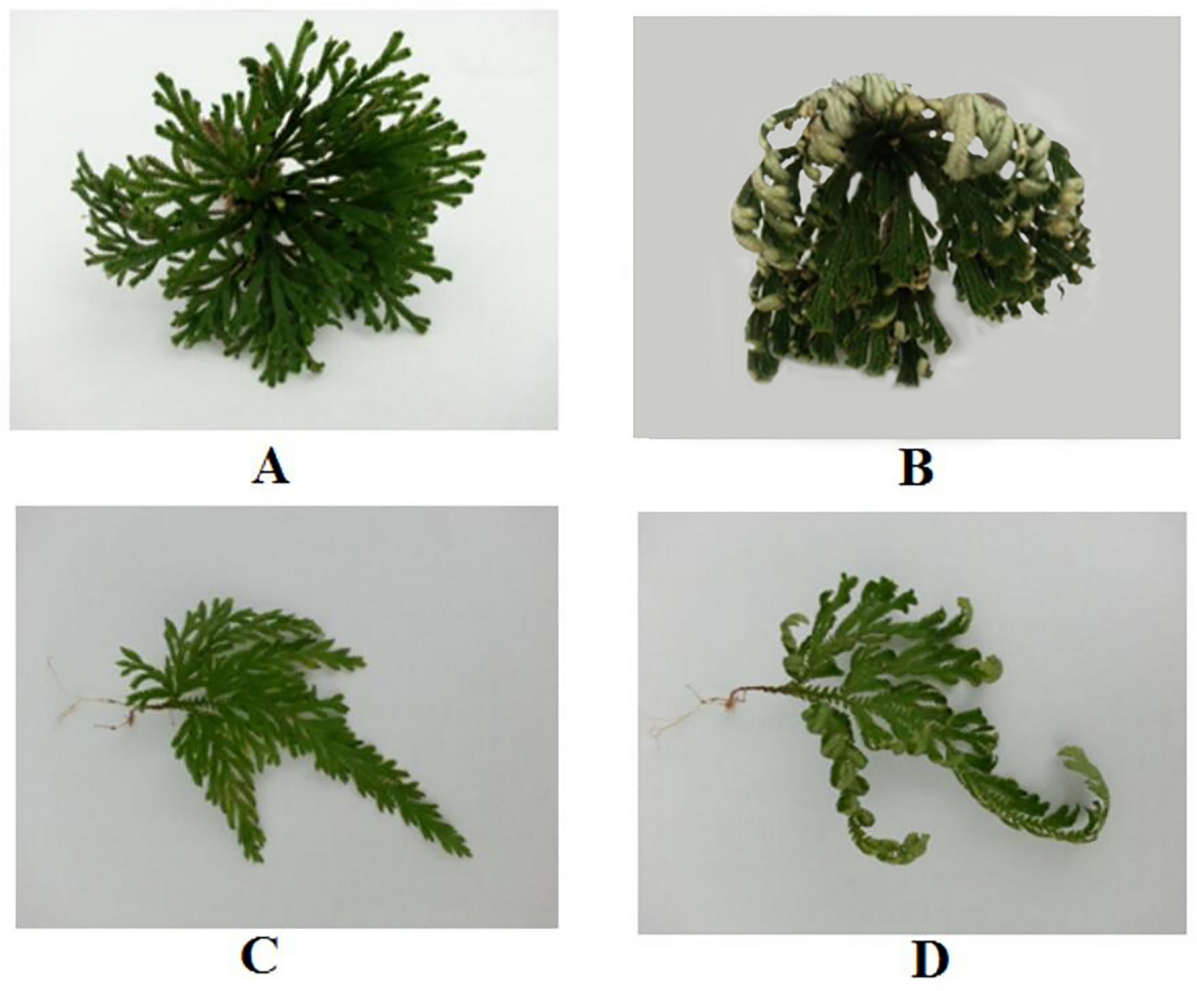
The plant morphology of *S*. *tamariscina* and *S*. *moellendorfii* at different relative water contents. A; *S*. *tamariscina* at a relative water content of 100%; B; *S*. *tamariscina* at a relative water content of 50%; C; *S*. *moellendorfii* at a relative water content of 100%; D; *S*. *moellendorfii* at a relative water content of 50%.

According to the methods described in previous studies[13,14], we established the rate of RWC loss during the dehydration process through the following steps. nine plants of each species were submerged in distilled water at room temperature and weighed every eight hours. After hydrated for 24 hours, plants of each species were fully hydrated and the weight of plants no longer changed. Then, the plants were incubated at 65 °C and weighed every two hours over a 24-h period to obtain dry weights. The RWCs of samples collected at each time point were calculated using the formula RWC (%) = (Fwt–Dwt)/(FTwt–Dwt) ·100%, where Fwt is the weight at any time point during the dehydration/rehydration cycle, Dwt is the weight after incubation at 65°C for 24 h, and FTwt is the weight after 24 h of hydration. This experiment was repeated for three times. To obtain materials at different relative water contents (RWCs), nine *S*. *tamariscina* and *S*. *moellendorffii* plants were treated according to the methods described above to collect samples of two RWCs (100% and 50%) at the corresponding time points. The samples were frozen and stored at –80 °C before RNA isolation. The plant morphology was shown in Fig 1.

In addition, *S*. *tamariscina* were treated according to the methods described above to collect samples at different times, including nine time points, before dehydration (t0); dehydration for 2 h (t2), 4 h (t4), 6 h (t6), and 12 h (t12); and rehydration for 3 h (f3), 6 h (f6), 12 h (f12), and 24 h (f24).

### 2.2 RNA isolation and illumina sequencing

RNA was isolated using an RNeasy Plus Mini Kit (#74134; Qiagen, Dusseldorf, Germany). The RNA integrity was monitored using 1% agarose gels. The RNA quality was measured using an Agilent 2100 Bioanalyzer (Agilent Technologies, Inc., Santa Clara, CA, USA).

The NEBNext^®^ Ultra^™^ RNA Library Prep Kit for Illumina^®^ (New England Biolabs (Beijing) Ltd., Beijing, China) was employed to generate sequencing libraries according to the manufacturer’s protocol. MRNA was enriched using poly-T oligonucleotide-attached magnetic beads. After the double-stranded cDNA was synthesized and purified, the 3′ ends of the DNA fragments were adenylated. The AMPure XP system was used to obtain fragments of 150–200 base pairs (bp). The prepared libraries were processed and analyzed using the Illumina Hiseq 2500 platform (Illumina, San Diego, CA, USA). The raw data of *S*. *tamariscina* and *S*. *moellendorfii* were submitted to the Short Read Archive database of National Center for Biotechnology Information (NCBI) under the project accession number SRR8149073 and SRR8149074, and in the fastq file format.

### 2.3 De novo assembly and functional annotation

The adapters and low-quality raw data (Q-value 20) were removed using Trimmomatic (version 0.30). After filtering, the quality was evaluated using FastQC (version 0.10.1). The clean data were assembled de novo using Trinity (version 2013-02-25) with three modules: Inchworm, chrysalis, and butterfly. The assembled contigs were clustered using Cluster Database at High Identity with Tolerance (CD-HIT) to obtain non-redundant unigene sequences. Transcripts of *S*. *tamariscina* and *S*. *moellendorfii* were mapped to the *S*. *tamariscina* whole genome assembly and the *S*. *moellendorffii* whole genome assembly using the Hisat2 software (http://ccb.jhu.edu/software/hisat2/index.shtml), respectively.

### 2.4 Differential expression analysis

RNA sequencing (RNA-Seq) using Expectation-Maximization (RSEM) was employed to estimate gene expression levels for Sm-50(*S*. *moellendorfii* grown in a RWC of 50%), Sm-100(*S*. *moellendorfii* grown in a RWC of 50%), St-50(*S*. *tamariscina* grown in a RWC of 50%), and St-100(*S*. *tamariscina* grown in a RWC of 100%). We used edgeR analysis, which is based on a negative binomial distribution model, to calculate the differential expression. Gene expression was estimated using the basemean and Hochberg values. According to the results of the edgeR analysis, a gene whose P-value was < 0.05 and had |log2fold-change| ≥ 1 was categorized as a DEG. In addition, GO enrichment and KEGG pathway enrichment analysis were carried out for the DEGs.

### 2.5 QRT-PCR analysis for validation of the transcriptome data

Ten DEGs were selected randomly for validation. The qRT-PCR reaction was performed on an ABI 7500 quantitative analyzer (Applied Biosystems, Foster City, CA, USA). The qRT-PCR reaction system contained 10 μL of qPCR SYBR Green SuperMixUDG, 2 μL of cDNA, 8.8 μL of dH2O, 0.4 μL of upstream PCR primer (0.2 μmol/L) and 0.4 μL of downstream PCR primer (0.2 μmol/L). The PCR cycling conditions were: 95 °C incubation for 10 min, followed by 30 cycles of 95 °C for 40 s and 60 °C for 1 min. The β-actin and GAPDH were used as internal control genes. The specific qRT-PCR primers (S1 Table) were designed and supplied by Sangon Biotech Co., Ltd. (Shanghai, China). The relative expression levels of the genes were calculated using the 2^-ΔΔCt (cycle threshold)^ method.

### 2.6 Validation of the integrated regulatory network of drought resistance

#### 2.6.1 Determination of trehalose and the activities of POD and CAT

*S*. *tamariscina* samples collected at different times (t0, t2, t4, t6, t12, f3, f6, f12, f24) were pulverized into fine powders. Five grams of sample powder was extracted with 50 ml of water in a water bath under 99 °C for 60 min. After filtration, the solution was collected and the volume was set to 50 ml by adding ethanol. The solution was centrifuged for 10 min at 10000 r/min and the supernatant was collected. The sample solution was made ready for HPLC analysis by filtering 1 ml of the supernatant through a 0.22-μm micro-porous membrane.

The Agilent 1100 series high performance liquid chromatography (HPLC) system (Agilent Technologies Inc., Santa Clara, CA, USA) and the Alltech 3300 Evaporative light scattering detector (Alltech Technologies Inc., USA) were employed to analyze the samples. The Prevail^™^ Carbohydrate ES sugar analytical column (250 mm × 4.6 mm, 5 mm; Shanghai Chenqiao Biosciences Co., Ltd.) was used and the mobile phase was acetonitrile/distilled water (65:35, v/v) at a flow rate of 0.8 ml/min. The column temperature was set at 25 °C and the injection volume of each sample was 20 μl. The temperature of the drift tube was 80 °C. The flow rate of nitrogen was 4.0 L/min. The gain was set at 10. The trehalose used as a reference substance was purchased from Shanghai Yuanye Biotechnology Co., Ltd (Shanghai, China).

The activities of POD and CAT were determined by UV spectrophotometry and guaiacol method, respectively. The above-mentioned experiments were repeated three times.

#### 2.6.2 QRT-PCR analysis to establish the change trends of resurrection genes expression levels

Fourteen genes (*ST27G60*, *ST225G21*, *ST441G130*, *ST48G551*, *ST212G10*, *ST57G70*, *ST378G1108*, *ST39G41*, *ST285G03*, *ST104G12*, *ST836G022*, *ST188G03*, *ST55G715*, and *ST75G63*) were selected from among the candidate resurrection genes involved in the integrated regulatory network. Expression levels of these genes in *S*. *tamariscina* samples collected at different times (t0, t2, t4, t6, t12, f3, f6, f12, f24) were analyzed using qRT-PCR. The reaction system, experimental equipment, and data processing methods were the same as those mentioned in section 2.5. The NADPH was used as an internal control gene. The specific qPCR primers were supplied by Sangon Biotech Co., Ltd. (S2 Table).

## Results

### 3.1 Transcriptional profiles

After sequencing, a large amount of clean data were obtained from *S*. *tamariscina* and *S*. *moellendorfii*, respectively. The detailed transcriptional pattern information is summarized in S3 Table. The Q20 scores were ≥ 99.49%, confirming the reliability of the sequencing results. Over 86% of the clean reads from *S*. *tamariscina* could be mapped to its genome sequence. Additionally, over 85% of the clean reads from *S*. *moellendorfii* were mapped to its genome sequence. The genome sequence of *S*. *moellendorffii* is available at http://genome.jgipsf.org/Selmo1/Selmo1.home.html [15,16]. The genome sequence of *S*. *tamariscina* is obtained from a previous study[17].

### 3.2 Classification Analysis of drought-inducible differentially expressed genes (DEGs)

#### 3.2.1 DEGs analysis of *S*. *tamariscina*

**Fig 2:**
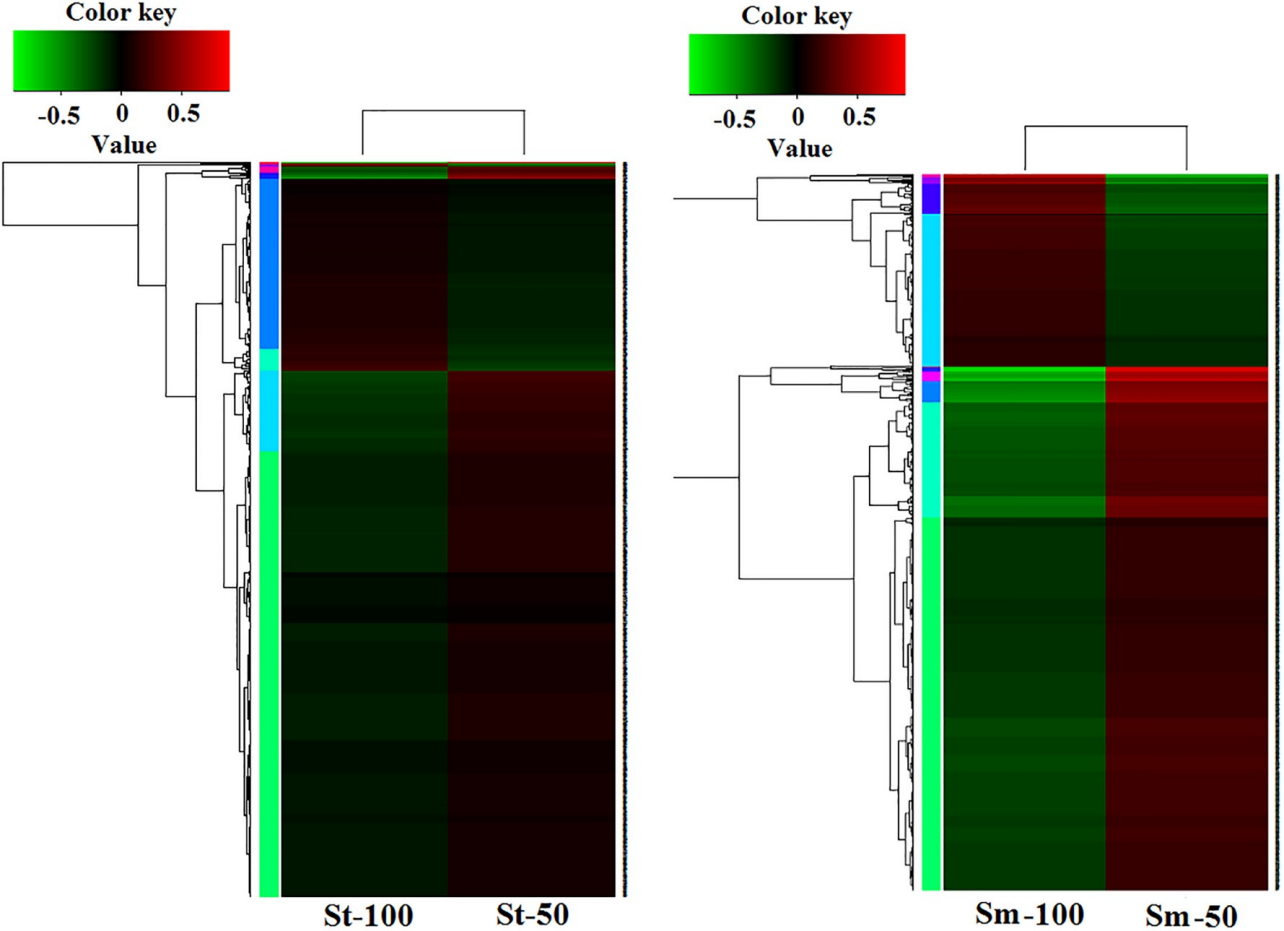
A heatmap of DEGs of *S*. *tamariscina* and *S*. *moellendorfii*. St-100, *S*. *tamariscina* at a RWC of 100%; St-50, *S*. *tamariscina* at a RWC of 50%; Sm-100, *S*. *moellendorfii* at a RWC of 100%; Sm-50, *S*. *moellendorfii* at a RWC of 50%.

In the comparison of St-100 versus St-50, 742 DEGs were identified, among which 546 were upregulated and 196 were downregulated (Fig 2). Obviously, more DEGs were overexpressed under drought conditions. The 742 DEGs were annotated in 46 GO subcategories, including 21 in biological process (BP), 13 in cellular component (CC), and 12 in molecular function (MF). Notably, we identified some interesting terms, such as antioxidant activity (GO:0016209), response to stimulus (GO:0050896), positive regulation of biological process (GO:0048518), and signaling (GO:0023052) among the DEGs.

The DEGs were also matched with the KEGG database, and the top 15 KEGG pathways are shown in S4 Table. Various significant pathways were observed to be associated with *S*. *tamariscina* drought tolerance, such as plant hormone signal transduction (ko04075), phenylalanine metabolism (ko00360), MAPK signaling pathway—plant (ko04016), and plant-pathogen interaction (ko04626).

#### 3.2.2 DEGs analysis of *S*. *moellendorffii*

In the comparison of Sm-100 versus Sm-50, 876 DEGs were identified, among which 641 unigenes were upregulated and 235 unigenes were downregulated (Fig 2). The 876 DEGs were annotated in 47 GO subcategories, including 22 in biological process (BP), 14 in cellular component (CC), and 11 in molecular function (MF). The DEGs were also matched with the KEGG database, and the top 10 KEGG pathways are shown in S5 Table.

#### 3.2.3 Comparative DEGs analysis between *S*. *tamariscina* and *S*. *moellendorffii*

**Fig 3:**
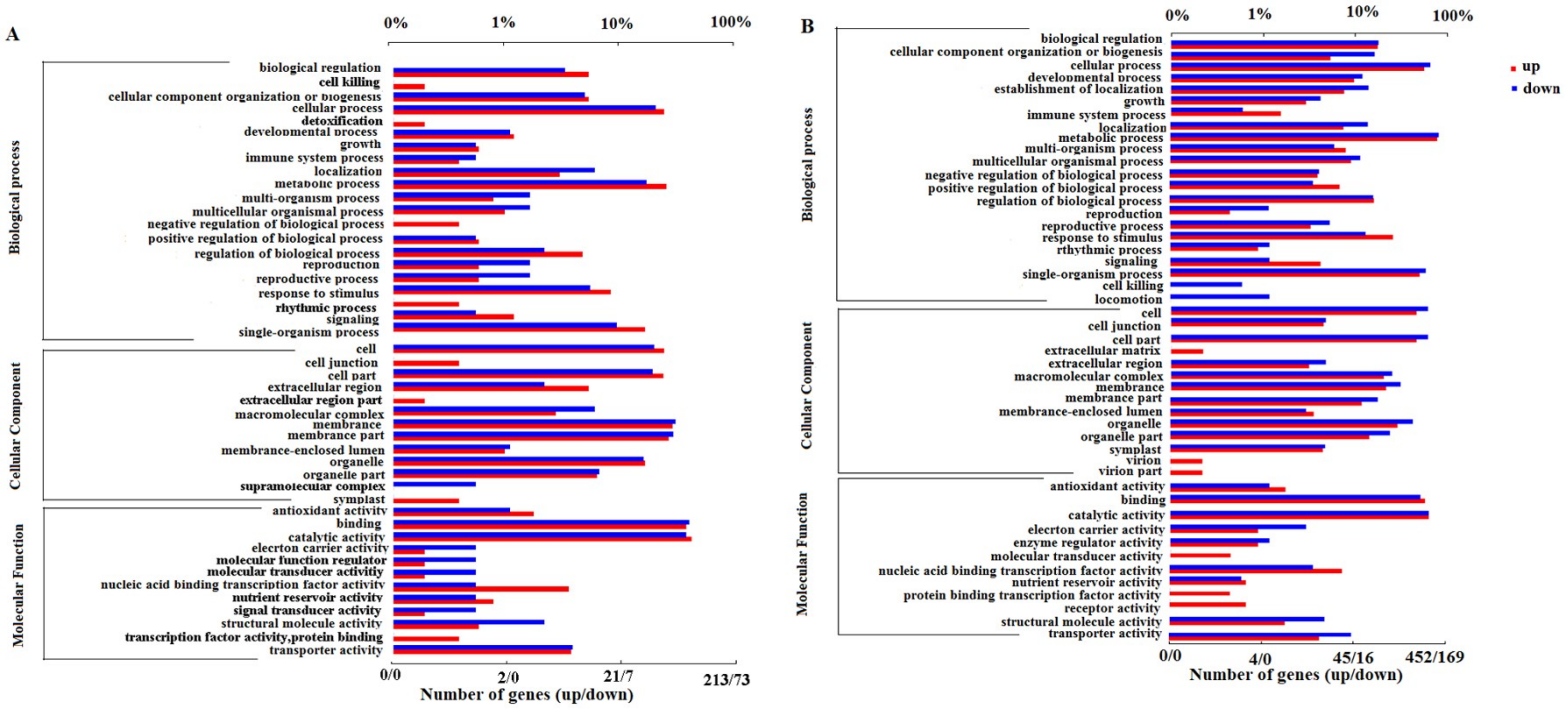
Go term classification of DEGs in *S*. *tamariscina* and *S*. *moellendorfii*. A; *S*. *tamariscina*; B, *S*. *moellendorfii*.

In *S*. *tamariscina* and *S*. *moellendorffii*, the major MF subcategories were “catalytic activity” (GO:0003824) and “binding” (GO:0005488). For BP, the most represented categories were “metabolic process” (GO:0008152), second was “cellular process” (GO:0009987). Finally, “cell” (GO:0005623) was the dominant subcategory among the CC terms. Phenylpropanoid biosynthesis (ko00940) pathway and Plant-pathogen interaction (ko 04626) pathway were annotated in both species. These results are listed in Fig 3.

In the comparison of the two species, we observed that the percentage of upregulated DEGs in *S*. *tamariscina was* a little higher than that in *S*. *moellendorffii*. More importantly, under drought conditions, many significant subcategories (e.g., transcription factor activity, signal transducer activity, molecular transducer activity, and detoxification) were unique in *S*. *tamariscina* but not in *S*. *moellendorffii*. And most genes of these subcategories were up-regulated.

### 3.3 Enrichment analysis of DEGs underlying drought stress

At a corrected P-value threshold of < 0.05, 896 DEGs of *S*. *tamariscina* were significantly enriched in 29 GO terms and 876 DEGs of *S*. *moellendorffii* were enriched in 63 GO terms. After comparing the 29 GO terms with the 63 GO terms, we noted that all 29 GO terms were only enriched in DEGs of *S*. *tamariscina*, while not enriched in *S*. *moellendorffii* DEGs. These 29 unique enriched GO terms were mainly associated with substance catabolic processes including L-phenylalanine catabolic process, erythrose 4-phosphate/phosphoenolpyruvate family amino acid catabolic process, xyloglucan metabolic process and organic acid catabolic process. 58 drought-responsive DEGs were provided from the 29 unique enriched GO terms and the expression level of these genes were almost up-regulated. The 58 genes may play pivotal roles in the drought resistance and considered as drought-inducible genes of *S*. *tamariscina*. The 29 unique enriched GO subcategories were summarized in S6 Table.

At a corrected P-value threshold of < 0.05, KEGG pathways significantly enriched in *S*. *tamariscina* and *S*. *moellendorffii* were summarized in Table 1. Photosynthesis (ko00195) and plant-pathogen interaction (ko04626) were identified in *S*. *moellendorffii*. Phenylalanine metabolism(ko00360) and phenylpropanoid biosynthesis(ko00940) pathways were identified in *S*. *tamariscina*. 31 genes associated with phenylalanine metabolism (ko00360) and phenylpropanoid biosynthesis (ko00940) pathways were considered as drought-inducible genes of *S*. *tamariscina*.

**Table 1:** Pathways significantly enriched in *S*. *tamariscina* and *S*. *moellendorffii* under drought stress.

No.	Pathway (Ko. number)	Genes (up-regulated/total)	species
1	Phenylalanine metabolism(ko00360)	17/18	St
2	Phenylpropanoid biosynthesis(ko00940)	28/31	St
3	Photosynthesis (ko00195)	2/16	Sm
4	plant-pathogen interaction (ko04626)	20/21	Sm

St, *Selaginella tamariscina*; Sm, *Selaginella moellendorfii*

### 3.4 Transcription factors (TF) analysis

In the present study, 12 TF families were differentially expressed in *S*. *tamariscina* and 19 TF families were differentially expressed in *S*. *moellendorffii* under drought conditions (S7 Table). Obviously, more TF families were differentially expressed under drought treatment in *S*. *moellendorffii*.

Nine TF families of *S*. *tamariscina* were common with those of *S*. *moellendorffii*. Among them, ethylene response factor (ERF) TFs (9 genes) was a major contributor. In addition, three TF families were annotated specially in *S*. *tamariscina*. including C2H2 (1 gene), HB (1 gene), and MYB-related (1gene) TFs. In total, 38 DEGs associated with TFs were obtained in *S*. *tamariscina* under drought.

### 3.5 Classification of drought-responsive genes in *S*. *tamariscina*

58 and 31 drought-responsive DEGs provided from the 29 unique enriched GO terms and 2 unique enriched KEGG pathways were considered as drought-inducible genes of *S*. *tamariscina*. After deleting the repeat genes of the 53 and 31 DEGs, we got 57 drought-responsive genes which were almost associated with the substance metabolic process. After searching the reported drought-relative genes in this study, other 76 DEGs were obtained as drought-relative genes in *S*. *tamariscina*.

Totally, 133 genes were divided into two major groups: functional genes (S8 Table) and regulatory genes (S9 Table). Functional genes encode proteins that are directly involved in many processes, such as cell protection and damage repair. Regulatory genes are implicated in signaling and transcriptional regulation [18].

#### 3.5.1 Analysis of drought responsive functional genes

In total, 72 DEGs were identified as related to functional processes in *S*. *tamariscina*. These 72 DEGS can be classified into four groups, including 35 DEGs associated with antioxidants, 13 DEGs related to cell wall modification, 15 DEGs keeping osmotic balance and others (9 DEGs).

Nine genes (*ST27G54*, 2.08; *ST48G79*, 1.80; *ST888G00*, 2.41; *ST962G054*, -6.32; *ST27G60*, 2.69; *ST27G52*, 1.65; *ST327G01*, 2.22; *ST659G04*, 2.52; *ST27G53*,1.38) were found to encode peroxidases (POD). In addition, catalase gene (*ST114G47*, 1.43), L-ascorbate oxidase gene (*ST84G12*, 1.34) and polyamine oxidase gene (*ST441G130*, *8*.*2*) were obtained. The expression levels of the above genes were upregulated except for that of *ST962G054* in *S*. *tamariscina*. The other 22 DEGs were associated with polyphenol synthesis, including genes encoding phenylalanine ammonia-lyase (*ST225G110*,2.04; *ST1032G08*, 2.96; *ST225G017*, 2.31; *ST208G326*, 8.85; *ST225G072*, 2.37; *ST225G27*, 2.89; *ST325G138*, 2.83; *ST225G098*, 3.07; *ST545G074*, 3.50; *ST737G03*, 3.05; *ST325G137*, 2.97; *ST225G21*, 2.31; *ST225G13*, 3.12; *ST208G327*, 2.89; *ST545G079*, -4.44), prephenate dehydratase (*ST57G46*, 1.66), 4-coumarate—CoA ligase (*ST2G06*, 6.60; *ST142G31*, 1.46; *ST4G332*, 1.42; *ST341G01*, 6.80) and cinnamoyl-CoA reductase 1(*ST215G05*, 1.87; *ST541G011*,1.73). A fatty acid desaturase gene was also upregulated in *S*. *tamariscina* under drought.

DEGs associated with cell wall modification were obtained, including genes involved in xyloglucan metabolic process (*ST141G06*, 2.23; *ST123G323*, 4.34; *ST365G01*, 3.24; *ST1113G02*, -5.86; *ST161G324*, 2.03; *ST161G39*, 1.81; *ST123G338*,2.33; *ST1G1055*, 1.40; *ST219G212*, 1.40), pectin metabolic process (*ST560G02*, 4.35; *ST533G02*, -9.93; *ST788G03*, 5.91) and cutin biosynthesis (*ST39G41*, 1.48). The ST39G41 gene encodes β-ketoacyl-CoA synthase, which increases the synthesis of ultra-long chain fatty acids and further synthesizes alkanes and aldehydes [19,20].

Eight DEGs involved in carbohydrate metabolism were identified, including three genes (*ST212G10*, 2.45; *ST134G05*, 2.24; *ST108G246*, 1.46) encoding chitinase, *ST57G70* (1.94) encoding galacturonosyltransferase, three genes (*ST378G1110*, 2.03; *ST378G1108*, 2.32; *ST1193G02*,2.20) encoding beta-amylase 1 and *ST42G06* (1.63) encoding trehalose-phosphate phosphatase. In addition, five genes (*ST593G00*, 1.93; *ST829G09*, 3.20; *ST48G511*, 1.57; *ST276G041*, 1.48; *ST48G571*, 1.50) were found to encode Late embryogenesis abundant protein (LEA). And two genes (*ST350G05*, 1.96; *ST688G01*, 2.12) encoding aquaporin protein were obtained. The expression levels of above fifteen genes related to osmotic balance were all upregulated.

#### 3.5.2 Analysis of drought responsive regulatory genes

In the present study, 61 DEGs were detected to be regulatory genes, including those encoding protein kinases (23), calcium sensors (3), TFs (26), hormone-related genes (8), and phospholipase (1).

Among the 23 genes encoding protein kinases, the serine/threonine kinase (STKs) genes were the dominant group with nine STKs genes upregulated and four STKs genes downregulated under drought conditions in *S*. *tamariscina*. In addition, six receptor-like protein kinase (RLKs), two leucine-rich repeat receptor-like protein kinase (LRR-RLK) and one upregulated mitogen-activated protein kinase kinase kinase (MAPKKK) genes were identified. The gene expression levels of major RLK were upregulated in *S*. *tamariscina* under drought conditions.

Calcium ions (Ca2+) are important second messengers involved in the signal transduction of the plant stress response, and calcium-binding proteins play an important role as calcium sensors in the plant calcium signal transduction pathway [21,22]. Two calcium-dependent protein kinase (CDPK) genes (*ST189G18*, 3.56; *ST112G33*, 2.03) were upregulated under drought conditions. In addition, one gene (*ST48G551*, 2.05) encoding calmodulin (CaM)-like proteins (CML) were also upregulated.

Twenty-six drought responsive TF genes were identified among the DEGs in *S*. *tamariscina*. They were classified into six major families: DREB (3), MYB (6), WRKY (2), NAC (4), ERF (10), and bZIP (1). In addition, eight DEGs were identified as hormone-related genes, which belonged to the ABA, gibberellins (GA) and Auxin (AUX) groups.

Phospholipid signaling has been reported to play an important role in the response to abiotic stress, such as drought, low temperature, and osmotic stress [23,24]. In the present study, one phospholipase (PLA) gene (*ST1G1230*, 1.89) were upregulated.

### 3.6 Quantitative real-time reverse transcription PCR (qRT-PCR) validation of DEGs from *S*. *tamariscina* and *S*. *moellendorffii*

To further validate the changes in gene expression observed in the differential expression analysis, qRT-PCR assays were performed on ten randomly selected DEGs. All 10 genes showed similar trends to those in the differential expression analysis (S1 Fig). Thus, we considered that the transcriptome-based DEG results were reliable to identify drought-responsive genes in the present study.

### 3.7 Validation of the candidate resurrection genes of an integrated regulatory network

**Fig 4:**
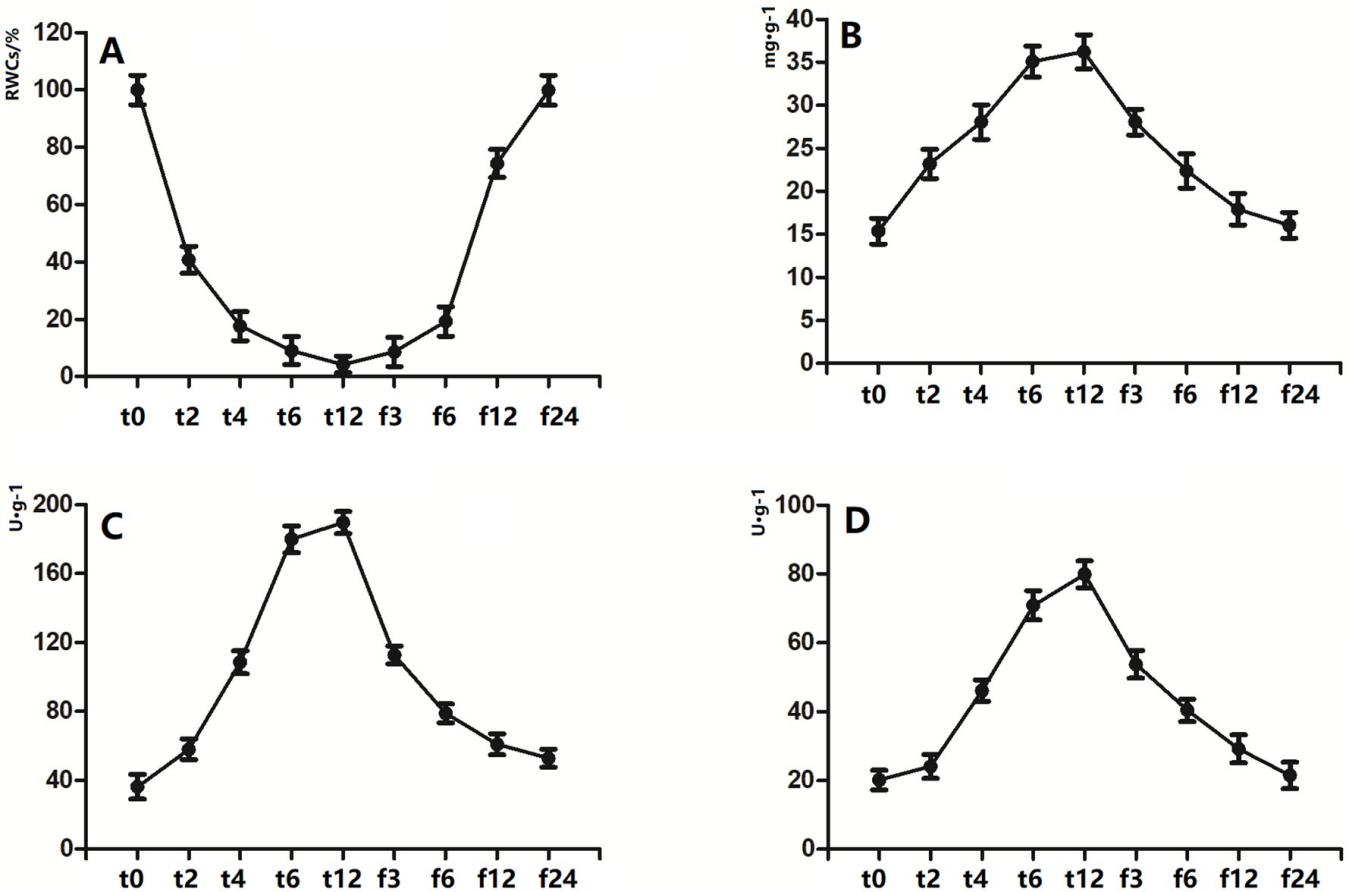
Determination of physiological indicators of *S*. *tamariscina* under different dehydration stress. A; the change of the RWC; B-C; the activities of POD, CAT; D; the content of trehalose.

The content of trehalose and the activities of peroxidase (POD) and catalase (CAT) of S. *tamariscina* samples collected at different times (before dehydration (t0); dehydration at 2 h (t2), 4 h (t4), 6 h (t6), and 12 h (t12); and rehydration 3 h (f3), 6 h (f6), 12 h (f12), and 24 h (f24)) showed similar change trends (Fig 4). The content of trehalose and the activities of POD and CAT gradually increased with the increase in dehydration time. The highest values were reached at 12 h of dehydration. As the rehydration time increased, the content of trehalose and the activities of POD and CAT gradually declined. After 24 hours of rehydration, the content of trehalose and activity of POD and CAT gradually returned to the original state.

**Fig 5:**
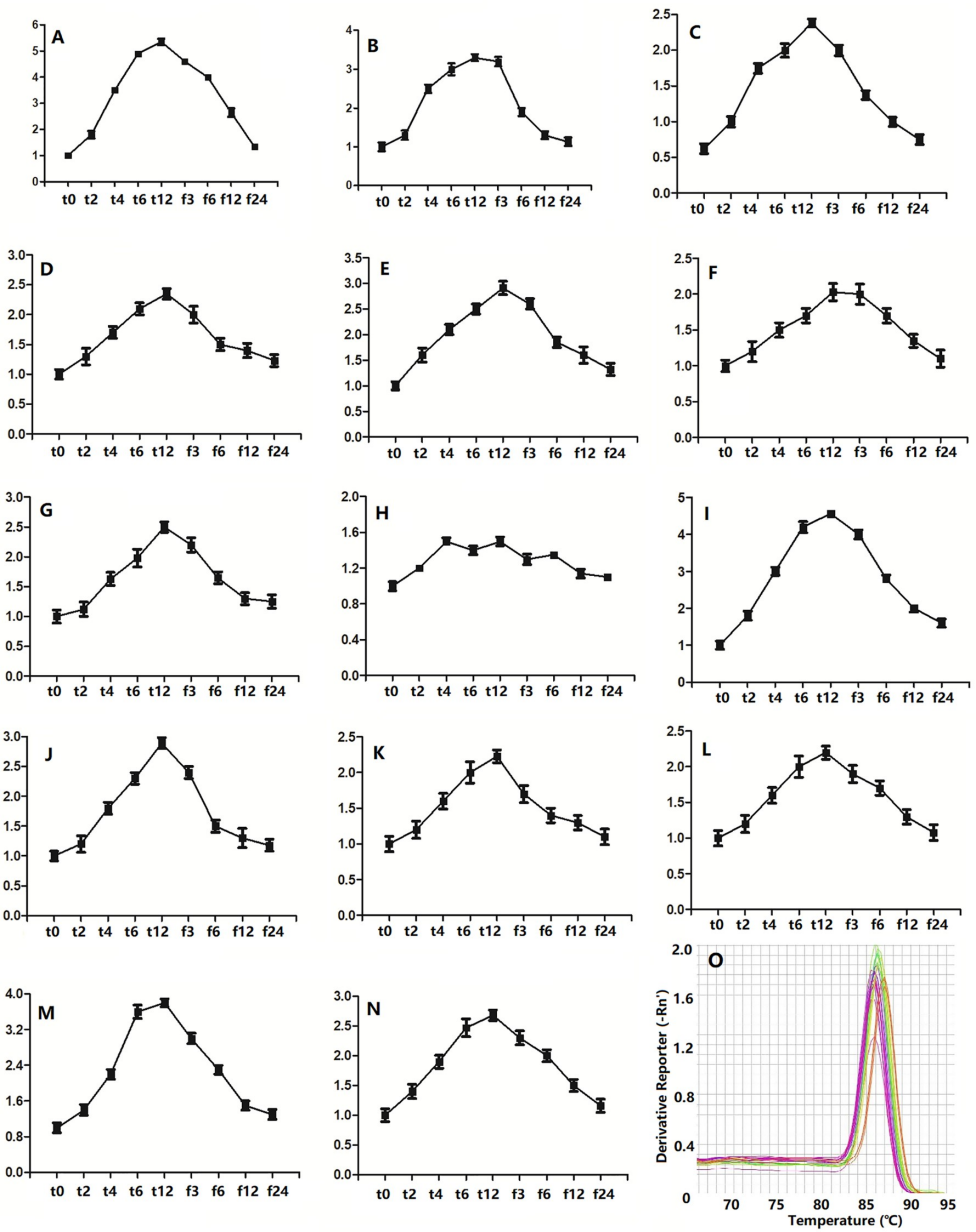
Gene expression profiles of *S*. *tamariscina* under different dehydration stress. A-N;Relative quantitation of the *ST27G60*, *ST225G21*, *ST441G130*, *ST48G551*, *ST212G10*, *ST57G70*, *ST378G1108*, *ST39G41*, *ST285G03*, *ST104G12*, *ST836G022*, *ST188G03*, *ST55G715*, *ST75G63*; O, Melting curve of PCR reaction.

In addition, the expression levels of 14 selected genes (*ST27G60*, *ST225G21*, *ST441G130*, *ST48G551*, *ST212G10*, *ST57G70*, *ST378G1108*, *ST39G41*, *ST285G03*, *ST104G12*, *ST836G022*, *ST188G03*, *ST55G715*, and *ST75G63*) showed similar trends to the content of trehalose and the activities of POD and CAT of *S*. *tamariscina* samples collected at different times (t0, t2, t4, t6, t12, f3, f6, f12, f24) (Fig 5).

## Discussion

**Fig 6:**
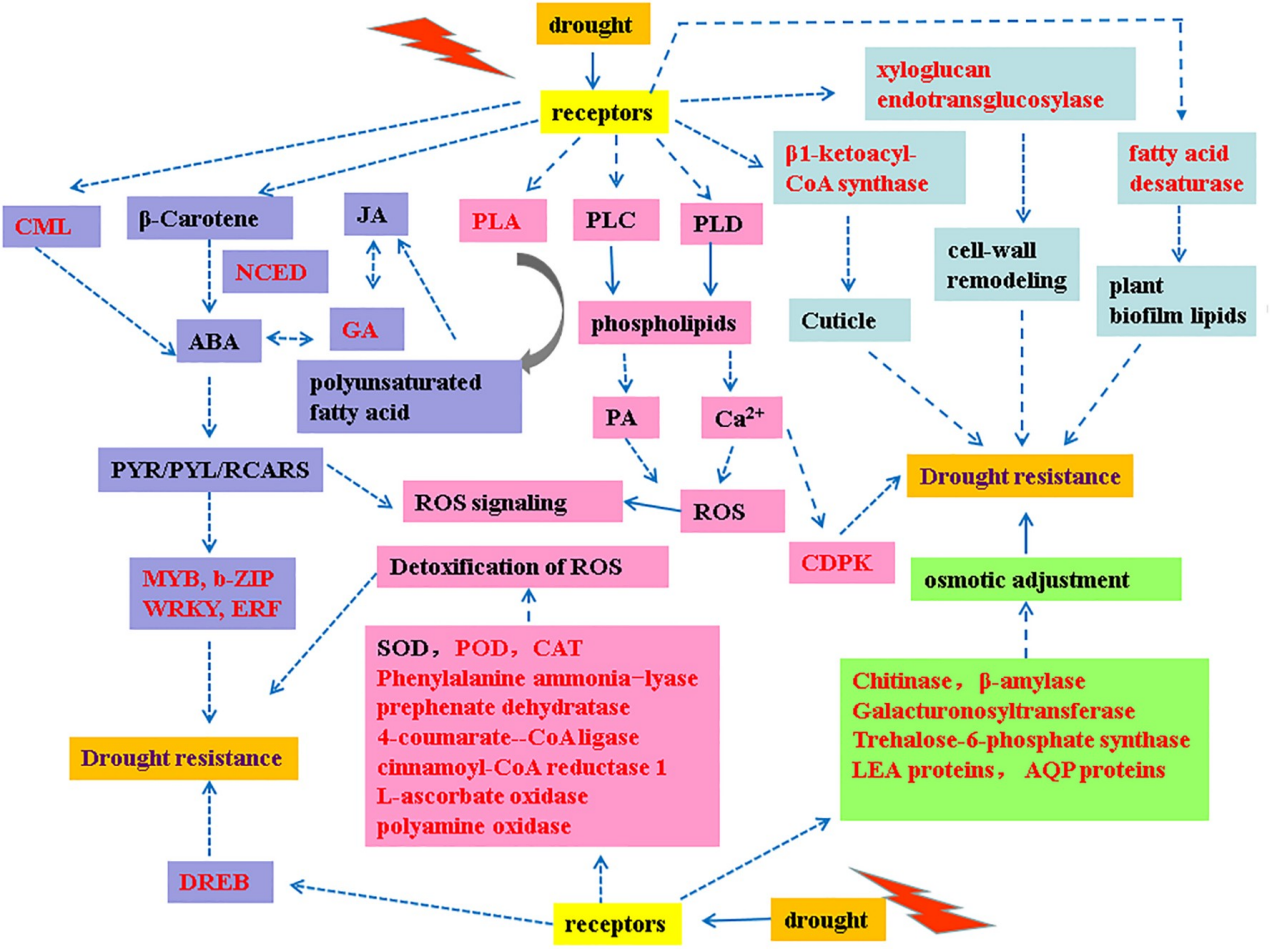
The comprehensive regulatory network of *S*. *tamariscina* under drought conditions.

*Selaginella tamariscina* (Beauv.) spring has received increasing attention recently because of its high medical value, special position in evolution, and outstanding drought tolerance. In the present study, a comparative transcriptional pattern analysis was conducted between *S*. *tamariscina* and *S*. *moellendorffii* to identify drought-related genes and to further clarify the molecular mechanism of drought tolerance in *S*. *tamariscina*. More upregulated DEGs than downregulated DEGs were identified in both *S*. *tamariscina* and *S*. *moellendorffii* under drought conditions. Among these DEGs, we identified a large number that are involved in several plant response processes under drought stress, including activation of anti-oxidation processes, osmotic adjustment, cuticle defense, and stress signal transduction. An integrated regulatory network of drought resistance was constructed to clarify the molecular mechanism of drought tolerance in *S*. *tamariscina* (Fig 6).

### 4.1 Activation of anti-oxidation processes

Increased levels of reactive oxygen species (ROS) will cause serious damage to normal plant cells. The present study highlighted the activation of anti-oxidation processes, indicating that *S*. *tamariscina* has developed antioxidant enzymes and antioxidants to reduce ROS-induced damage.

First, expression levels of most genes encoding peroxidases and catalase were upregulated, indicating the antioxidant enzymes activities were triggered to reduce oxidative damage. Second, four important enzymes, phenylalanine ammonia-lyase, prephenate dehydratase,4-coumarate—CoA ligase, cinnamoyl-CoA reductase 1 were almost upregulated, suggesting that these enzymes and their associated pathways play important roles in protecting *S*. *tamariscina* from drought stress, which is consistent with the results of a previous study [25]. The above four enzymes were all involved in polyphenol synthesis and metabolism. Polyphenols were low molecular weight antioxidants which can effectively scavenge harmful radicals and stabilize lipid oxidation[26]. The upregulated gene expression of enzymes involved in polyphenol synthesis and metabolism demonstrated the importance anti-oxidation role of polyphenol in *S*. *tamariscina* under drought. Fifteen genes were annotated as genes encoding phenylalanine ammonia-lyase which acts as a key enzyme involved in the biosynthesis of isoperonoid antioxidative compounds and a key regulator in phenylalanine metabolism. Phenylalanine metabolism has been reported to be an alternative pathway to enhance the antioxidant capacity in plants [27]. Therefore, we hypothesized that *ST225G110*, *ST1032G08*, *ST225G017*, *ST208G326*, *ST225G072*, *ST225G27*, *ST325G138*, *ST225G098*, *ST545G074*, *ST737G03*, *ST325G137*, *ST225G21*, *ST225G13*, *ST208G327*, and *ST545G079* are key drought responsive genes in *S*. *tamariscina*. Third, one L-ascorbate oxidase gene was upregulated. L-ascorbate oxidase has high affinity for H2O2 and ascorbate, which suggests that ascorbate oxidase can detoxify H2O2 to reduce the drought damage and increase drought tolerance[28]. Polyamine catabolism has also been reported to regulate ROS accumulation and ROS-scavenging enzymes in cells[29]. Interestingly, a polyamine oxidase (POX) gene (*ST441G130*, 8.2) in polyamine catabolism was found in our study and the expression levels of *ST441G130* was markedly increased, which demonstrated that *S*. *tamariscina* can alleviate drought-induced ROS damage via polyamine catabolism.

In addition, the Ca2+ (calmodulin (CaM)-dependent) signaling pathway is associated with the regulation of ROS accumulation and its elimination [30]. One CaM-like proteins (CML) were also upregulated. It was reported that ShCML44 in wild tomato could regulate the clearance of ROS to prevent the oxidative damage caused by their excessive accumulation under drought stress [31].

### 4.2 Osmotic adjustment

To maintain a cell’s water absorption ability and normal structure under drought stress, plants need to accumulate soluble sugars and proline, which are defined as osmoprotectants [31]. In the present study, many DEGs involved in soluble carbohydrate metabolism were identified.

Chitinase and alpha-1,4-galacturonosyltransferase in amino sugar and nucleotide sugar metabolism (ko00520) showed significantly increased expression in *S*. *tamariscina* under drought conditions. A chitinase-like protein was overexpressed in the Arabidopsis hot2 mutant, which has excellent tolerance to heat, salt, and drought stresses [32]. Galacturonosyl transferase is the core element of pectin biosynthesis in the plant cell wall, indicating that upregulation of alpha-1,4-galacturonosyltransferase could adjust the osmotic potential via alterations to the plant cell wall [33]. Significant upregulation of genes encoding beta-amylase, which are involved in starch and sucrose metabolism (ko00500) was also noted. Starch is a major form of carbon storage in plants, and mobilization of starch reserves is important for survival and recovery from stresses such as drought. Beta-amylase catalyzes the breakdown of starch into soluble sugars[34]. The expression levels of beta-amylase genes (*ST378G1110*, *ST378G1108* and *ST1193G02*) were upregulated under water stress in the present study, indicating that beta-amylase function in the accumulation of soluble sugars in *S*. *tamariscina* under drought stress. Trehalose-6-phosphate synthase (TPS) is a key enzyme in the synthesis of trehalose in plants[35]. Trehalose was a stable non-reducing disaccharide composed of two glucoses and was reported to play an important role in the drought resistance of plants[36–38]. In present study, an upregulated trehalose-phosphate phosphatase gene (*ST42G06*, 1.63) was obtained.

In addition, Late embryogenesis abundant protein (LEA) has osmo-protective effect[39], and 5 LEA genes are significantly upregulated in *S*. *tamariscina* under drought. Previous studies have confirmed that aquaporin protein (AQP) can transport water and other small molecules through biological membranes, which is crucial for plants to combat stress caused by drought[40]. Overexpression of the wheat aquaporin gene, TaAQP7 can enhance drought tolerance in transgenic tobacco[41,42]. Two AQP genes in present study were all upregulated, indicating that *S*. *tamariscina* is able to enhance water absorption capacity of fibrous roots, and to maintain water adapt to arid environment.

### 4.3 Cuticle defense

Under drought stress, the leaves of *S*. *tamariscina* curled, and when re-watering, the leaves gradually stretch to normal state. This plant morphology change indicated that cuticle defense strategies were employed to resist drought damage.

Firstly, the cuticle is composed of an inner layer and an outer layer of wax that can protect plants against water loss [43]. DEG involved in cutin biosynthesis (*ST39G41*, 1.48) was identified in *S*. *tamariscina*. The *ST39G41* gene encodes β-ketoacyl-CoA synthase, which increases the synthesis of ultra-long chain fatty acids and further synthesizes alkanes and aldehydes. Increased alkanes and aldehydes in the cuticle wax can enhance plant drought resistance [20,44]. Secondly, genes encoding xyloglucan endotransglucosylase/hydrolase (XTHs) and xyloglucan alpha-1,6-glycosyltransferase were obtained. XTHs have been reported to participate in cell-wall elongation and reconstruction through the cleavage and reformation of bonds between xyloglucan chains[45–47]. The up-regulated expression levels of XTHs genes indicated that *S*. *tamariscina* may reduce transpirational water loss in response to dehydration stress by increased cell-wall remodeling activity of XTHs. Thirdly, fatty acid desaturase (FAD) is a key enzyme for the formation of unsaturated fatty acids which are the main structural components of plant biofilm lipids[48]. Studies have shown that overexpression of BnFAD3 or AtFAD8 genes in tobacco increases the content of unsaturated fatty acids in membrane lipids, thereby avoiding membrane lipid peroxidation caused by excessive ROS and enhancing the drought resistance of plants[49]. In present study, a FAD gene (*ST104G12*, 1.67) was upregulated, indicating that changing the structure of membrane lipids and avoiding membrane lipid peroxidation was an important way for *S*. *tamariscina* to resist drought.

### 4.4 Signal transduction

We observed that several responses were triggered under drought stress, indicating that numerous signal transduction processes and complex pathways are employed to combat drought stress in *S*. *tamariscina*.

Receptor kinases are the bridge between the perception of stress and signal transduction. Most leucine-rich repeat receptor-like protein kinase (LRR-RLK) genes were downregulated under drought stress in *S*. *tamariscina*. An LRR-RLK gene in rice serves as a negative regulator under drought, which is consistent with our observations [50]. RLKs play important roles in perceiving extracellular stimuli and activating downstream signaling responses [51]. Four upregulated RLKs were identified in our data. The expression levels of unigenes encoding mitogen-activated protein kinases (MAPKs), and serine-threonine kinases (STKs) varied under drought stress in *S*. *tamariscina*. As important members of the MAPK cascade, MAPKKKs often play key roles in plant responses to stress. Ning et al. noted that the expression levels of seven selected MAPKKK genes were obviously upregulated by several stress treatments, which was consistent with the results of our study [52,53]. Td4IN2 is a drought-responsive gene that encodes a resistance-like protein with serine/threonine protein kinase activity [54]. The changes in the expression levels of major STKs observed in this study could also be the result of drought-responsive gene activation.

Drought stress causes an increase in the intracellular Ca2+ concentration, which leads to increased calcium-dependent protein kinase (CDPK) gene expression to promote the downward transmission of drought signals [55]. Two significantly upregulated CDPK genes among our DEGs confirmed the involvement of Ca2+ signaling.

Transcription factors are often involved in plant responses to drought. The *ST285G03*, *ST518G032*, *ST7G1628*, *ST34G424*, *ST494G08*, *ST58G91*, *ST44G415*, *ST565G040*, *ST131G35* and *ST1068G022* genes, which encode ERFs, were all upregulated under drought stress. Studies have confirmed that overexpression of ERFs can increase osmotic stress, oxidation, and superoxide dismutase gene expression, and increase photosynthetic carbon assimilation/metabolism to significantly enhance the drought resistance of tobacco[56]. In addition, *ST188G03*, *ST429G11* and *ST170G21* encode dehydration-responsive element-binding protein 3 (DREB3), DREB2A and DREB1F, respectively. DREB3 and DREB2A specifically interact with cis-acting dehydration-responsive element/C-repeat (DRE/CRT) involved in drought stress-responsive gene expression in *Arabidopsis thaliana*[57,58]. MYB TFs play a specific role in response to water stress, such as the regulation of stomatal movement, the control of suberin and cuticular waxes synthesis and the regulation of flower development [59]. In present study, six gene associated with MYB were obtained and the expression levels of these MYB genes were all upregulated.

The key enzyme in ABA synthesis is 9-cis-epoxycarotenoiddioxygenase (NCED). When subjected to the stress of an arid environment, the expression of ABA synthase was rapidly increased and downstream transcription factors (MYB, b-ZIP, WRKY, ERF) were activated. Gene *ST55G715* encodes NCED and was significantly induced under drought, confirming that drought conditions enhance the synthesis of ABA. The rapid accumulation of ABA promotes stomatal closure, thereby reducing the water loss caused by water transpiration [60]. In addition, ABA further inhibits the activity of phosphatase 2C (PP2C) after binding to its receptor, PYR/PYL/RCAR, to release SnRK2's autophosphorylation and protein phosphorylation activity, which ultimately activates downstream ABA-dependent TFs, such as MYB factors [61]. In addition, three DEGs associated with GA signals were also upregulated. GA can interact with the ABA signaling pathway to form a complex regulatory network. DELLA and XERICO proteins are important links to the GA, ABA, and jasmonic acid (JA) signaling pathways [62].

### 4.5 Validation of the candidate resurrection genes of the integrated regulatory network

The change in RWC is an important indicator that reflects the drought resistance of plants. The RWC of *S*. *tamariscina* declined to 4.24% after dehydration for 12 h. While the RWC of *S*. *tamariscina* rehydrated for 24 h reached to 99.89%, returning to the RWC level of *S*. *tamariscina* before dehydration. (Fig 4). This indicated that *S*. *tamariscina* could be restored to its original state upon resurrection.

As the RWC decreased under drought stress, the anti-oxidation system was strongly activated. The gene expression of *ST27G60* encoding POD increased gradually as the dehydration time increased, reaching its highest level after dehydration for 12 h. Meanwhile the content of POD and CAT in *S*. *tamariscina* dehydrated for 12 h also reached to a maximum value. In addition, the expression of *ST225G21* (encoding phenylalanine ammonia-lyase), *ST441G130* (encoding polyamine oxidase), and *ST48G551* (encoding CaM-like proteins) showed similar change trends to that of *ST27G60*, confirming that *S*. *tamariscina* has developed antioxidant enzymes and antioxidants to reduce ROS-induced damage.

The gene expression of *ST212G10* (encoding chitinase), *ST57G70* (encoding alpha-1,4-galacturonosyltransferase), and *ST378G1108* (encoding beta-amylase) increased gradually as the dehydration time increased and declined gradually with increased rehydration time. This signified that increased expression of these genes could increase the content of soluble carbohydrates to maintain high intracellular osmotic pressure to reducing water loss from *S*. *tamariscina* under drought conditions. Moreover, the trehalose content increased gradually during the dehydration process and returned to its origin level in *S*. *tamariscina* after dehydration. Trehalose can reduce the damage caused by drought stress in three ways. First, trehalose can substitute water to interact with biomolecules to maintain normal knots of protein structure and fluidity of the lipid bilayer. Second, trehalose can maintain the biological structure and function of biomolecules in by acting as a vitrifying agent. Third, trehalose can preferentially combine with the water molecules on the surface of the protein to reduce the solvation layer radius of the protein and stabilize its molecular structure, which is beneficial to resist the effects of drought and other adverse environmental conditions [63,64].

The gene expression of *ST39G41* (encoding proteins involved in cutin biosynthesis), and *ST104G12* (encoding FAD) also increased gradually as the dehydration time increased and declined gradually with increasing rehydration time. This implied that physiological responses, especially cuticle defense, were activated in *S*. *tamariscina* under drought and that these genes were resurrection genes of *S*. *tamariscina*. In addition, the trends of gene expression of *ST836G022*(encoding RPK1), *ST285G03* (encoding ERF), *ST188G03* (encoding DREB3), *ST55G715* (encoding NCED), and *ST75G63* (encoding GA) were similar to those of *ST39G41*, and *ST104G12*. These results showed that numerous signal transduction processes and complex pathways are employed to combat drought stress in *S*. *tamariscina*.

In summary, activation of anti-oxidation processes, osmotic adjustment, cuticle defense and signal transduction form an integrated regulatory network of drought resistance in *S*. *tamariscina*.

## Supporting information

S1 Fig QRT-PCR validation of DEGs from *S*. *tamariscina* and *S*. *moellendorffii*. St-100, *S*. *tamariscina* at a RWC of 100%; St-50, *S*. *tamariscina* at a RWC of 50%; Sm-100, *S*. *moellendorfii* at a RWC of 100%; Sm-50, *S*. *moellendorfii* at a RWC of 50%.
(TIF)

S1 Table List of primer sequences used for qRT-PCR validation. (DOCX)

S2 Table List of primer sequences used to establish the change trends of resurrection genes expression levels. (DOCX)

S3 Table Illumina RNA sequencing of *S*. *tamariscina* and *S*. *moellendorfii*. (DOCX)

S4 Table The top 15 KEGG pathways in *S*. *tamariscina*. (XLS)

S5 Table The top 10 KEGG pathways in *S*. *moellendorffii*. (XLSX)

S6 Table Unigenes in 29 GO terms that only enriched in *S*. *tamariscina*. (XLS)

S7 Table Different expressed transcription factors in *S*. *tamariscina*. (XLSX)

S8 Table Classification of drought responsive functional genes in *S*. *tamariscina*. (XLS)

S9 Table Classification of drought responsive regulatory genes in *S*. *tamariscina*. (XLS)
