# Supplementary figures and images for: Identifying resurrection genes through the differentially expressed genes between Selaginella tamariscina (Beauv.) spring and Selaginella moellendorffii Hieron under drought stress
Source: PLoS One. 2019 Nov 13;14(11):e0224765. doi: 10.1371/journal.pone.0224765 (PMC6853609; doi:10.1371/journal.pone.0224765)

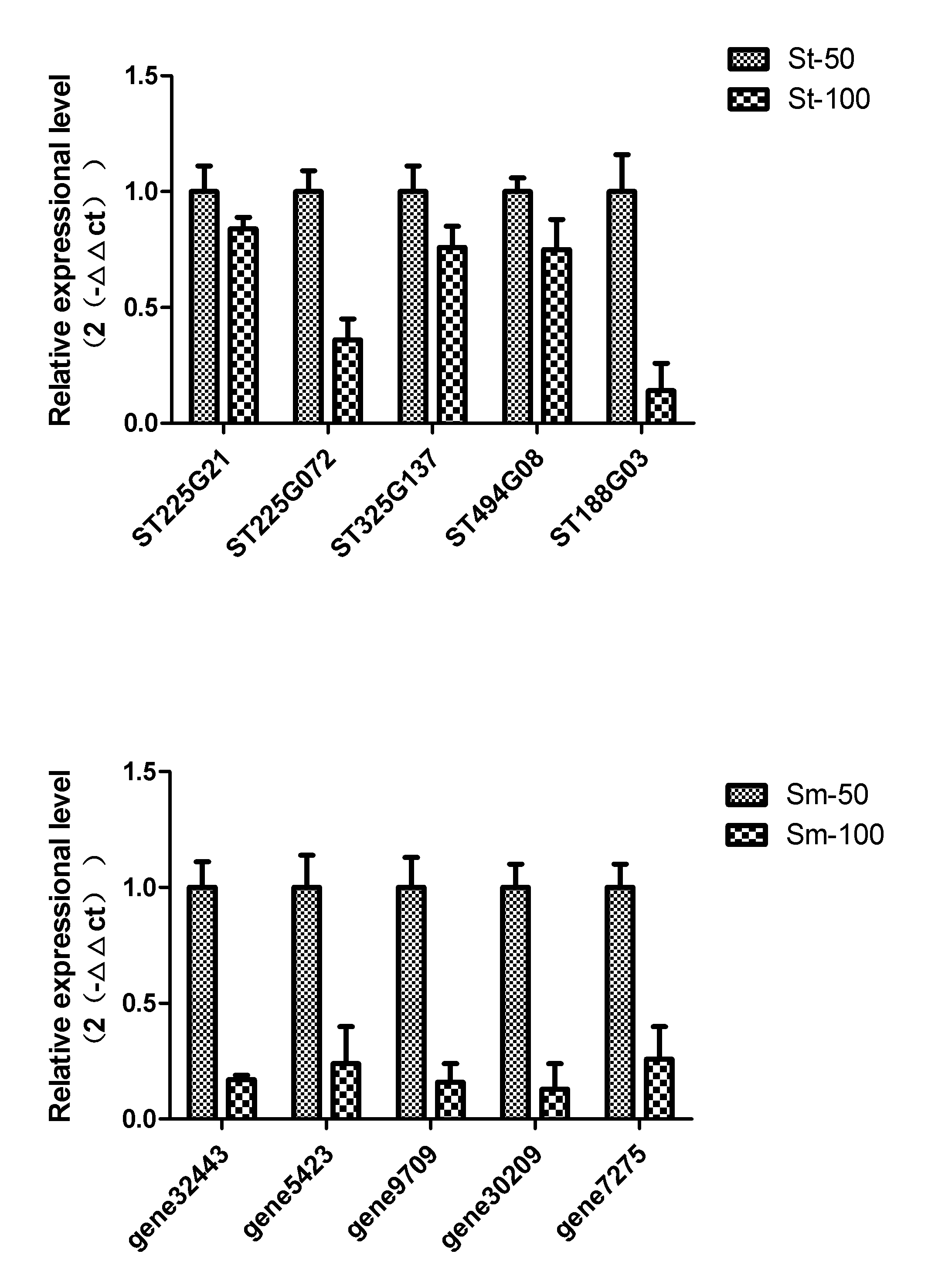

Supplement: S1 Fig — St-100, S. tamariscina at a RWC of 100%; St-50, S. tamariscina at a RWC of 50%; Sm-100, S. moellendorfii at a RWC of 100%; Sm-50, S. moellendorfii at a RWC of 50%. (TIF) [file pone.0224765.s001.tif]
