# Supplementary material for: Identifying resurrection genes through the differentially expressed genes between Selaginella tamariscina (Beauv.) spring and Selaginella moellendorffii Hieron under drought stress
Source: PLoS One. 2019 Nov 13;14(11):e0224765. doi: 10.1371/journal.pone.0224765 (PMC6853609; doi:10.1371/journal.pone.0224765)
Supplement: S1 Table — (DOCX) [file pone.0224765.s002.docx]

S1 Table. List of Primer sequences used for qRT-PCR validation

| Primer | Direction | Nucleotide Sequence (5’ → 3’) |
| --- | --- | --- |
| ST225G21-F | Forward | CCTCCTCAAACAAGCCGA |
| ST225G21-R | Reverse | GAGCACATGCAAGCCACA |
| ST225G072-F | Forward | CGTCTTCTCTTCTTTCTC |
| ST225G072-R | Reverse | CATGTCTTCACGTACATC |
| ST325G137-F | Forward | TTGTGACTCTGGCTTGGGA |
| ST325G137-R | Reverse | GCATTGGACTGATCGTTGG |
| ST494G08-F | Forward | TTCGTTCGGAAAGTTGAG |
| ST494G08-R | Reverse | CGAGAAGAGAGTGTGGGTC |
| ST188G03-F | Forward | CTCTCTCCTTCCTCGCTC |
| ST188G03-R | Reverse | TGGTCTTCGTCTTGCTCA |
| Gene32443-F | Forward | AGTTTCGTGGGGTGCGG |
| Gene32443-R | Reverse | GGGAAGTTGAGCTTGGCG |
| Gene5423-F | Forward | GCAAGTGGGATGGTGTAA |
| Gene5423-R | Reverse | AGCCGAGTCAAGTGTGG |
| Gene9709-F | Forward | AGGTGAAGATACTGGACGC |
| Gene9709 -R | Reverse | GAAACGCAGGAATAAGCC |
| Gene30209-F | Forward | ATCGTCCGGGAAGTTGAG |
| Gene30209-R | Reverse | TGAAGCAGGCCAGAGTGTG |
| Gene7275-F | Forward | GGAGAGCCTGGATTGCCC |
| Gene7275-R | Reverse | GAAGGATTCCCCGCGTGT |
| GAPDH-F | Forward | GACAGGCACAATAGGACC |
| GAPDH-R | Reversed | GGAATACAAGAGGGAAGG |
| β-Actin-F | Forward | AACTATGTCCCAATCTGCG |
| β-Actin-R | Reversed | TTTCTGCCATTCTTCCTGT |
