# Supplementary material for: Identifying resurrection genes through the differentially expressed genes between Selaginella tamariscina (Beauv.) spring and Selaginella moellendorffii Hieron under drought stress
Source: PLoS One. 2019 Nov 13;14(11):e0224765. doi: 10.1371/journal.pone.0224765 (PMC6853609; doi:10.1371/journal.pone.0224765)
Supplement: S2 Table — (DOCX) [file pone.0224765.s003.docx]

S2 Table. List of Primer sequences used to establish the change trends of resurrection genes expression levels

| Primer | Direction | Nucleotide Sequence (5’ → 3’) |
| --- | --- | --- |
| ST27G60-F | Forward | GCCACTGTTGAAAACTCTTGC |
| ST27G60-R | Reverse | GCTATTTGCCCCTCCTTGA |
| ST225G21-F | Forward | GTCATTCTCCTCACCCAT |
| ST225G21-R | Reverse | ACACTCACAACTGCTCCC |
| ST441G130-F | Forward | ATGCAGACGATCGTCTCGAGGCATG |
| ST441G130-R | Reverse | CATGCCTCGAGACGATCGTCTGCAT |
| ST48G551-F | Forward | CGAGCCCGACTAAGACCCT |
| ST48G551-R | Reverse | GACGGATTCATCAGCTTGGAG |
| ST212G10-F | Forward | GGGAACTTACCCAGCAGACA |
| ST212G10-R | Reverse | CCAGAAGAAATAACCCCTCAAA |
| ST57G70-F | Forward | GGTTGGAGACACGGTGAG |
| ST57G70-R | Reverse | CTGCCATTGTAGAAGTGAAGAG |
| ST378G1108-F | Forward | CTTACGAACCGATGATTAC |
| ST378G1108-R | Reverse | CTCCCAAGGCTGCTCTAC |
| ST39G41-F | Forward | TAGAAATCACTCCAGCAC |
| ST39G41-R | Reverse | CTACGCCTTCATTATCAG |
| ST285G03-F | Forward | CAGACGAGGATATGGTTAGGG |
| ST285G03-R | Reverse | CATCGGAGTAGCAGGGAGA |
| ST104G12-F | Forward | GTGTTTGGGTTGGCTGCTG |
| ST104G12-R | Reverse | CGTCCGATGGCTTATCCT |
| ST836G022-F | Forward | ACATCCCAGGAGGACAAA |
| ST836G022-R | Reversed | GACTGCATTCAAACCCATA |
| ST188G03-F | Forward | GGAACGAGCCTAACCAAA |
| ST188G03-R | Reverse | CTGTCTCCCAGCAGCATT |
| ST55G715_g1-F | Forward | CAAGTTGAGCCTTATTTCG |
| ST55G715-R | Reverse | GTCCCTGACTGTTTCTGTTT |
| ST75G63-F | Forward | TCTATGCCTCCGTCTGTG |
| ST75G63-R | Reverse | TCTTTCTGCTCGTATGGTA |
| NADPH-F | Forward | GACAGGCACAATAGGACC |
| NADPH-R | Reversed | GGAATACAAGAGGGAAGG |
