# Supplementary material for: Identifying resurrection genes through the differentially expressed genes between Selaginella tamariscina (Beauv.) spring and Selaginella moellendorffii Hieron under drought stress
Source: PLoS One. 2019 Nov 13;14(11):e0224765. doi: 10.1371/journal.pone.0224765 (PMC6853609; doi:10.1371/journal.pone.0224765)
Supplement: S3 Table — (DOCX) [file pone.0224765.s004.docx]

S3 Table. Illumina RNA sequencing of *S. tamariscina* and *S.* *moellendorfii*

| Data type | Samples | Total_Reads | Total_Bases | Q20% | GC% |
| --- | --- | --- | --- | --- | --- |
| Raw data | St-100 | 43386430 | 4233714537 | 99.55 | 48.20 |
| Raw data | St-50 | 60324910 | 5883453773 | 99.57 | 48.21 |
| Raw data | Sm-100 | 34610686 | 3340344727 | 99.49 | 53.18 |
| Raw data | Sm-50 | 49018052 | 4724792570 | 99.49 | 53.09 |
| Clean data | St-100 | 42556786 | 4182973271 | 99.60 | 48.19 |
| Clean data | St-50 | 59108160 | 5807164961 | 99.61 | 48.21 |
| Clean data | Sm-100 | 33802842 | 3292526653 | 99.54 | 53.18 |
| Clean data | Sm-50 | 47806018 | 4654922977 | 99.53 | 53.09 |
